# The social buffering of pain by affective touch: a laser-evoked potential study in romantic couples

**DOI:** 10.1093/scan/nsy085

**Published:** 2018-09-24

**Authors:** Mariana von Mohr, Charlotte Krahé, Brianna Beck, Aikaterini Fotopoulou

**Affiliations:** 1 Research Department of Clinical, Educational and Health Psychology, University College London, London, UK; 2 Department of Psychology, Institute of Psychiatry, Psychology, and Neuroscience, King’s College London, London, UK; 3 Institute of Cognitive Neuroscience, University College London, London, UK

**Keywords:** affective touch, attachment style, laser-evoked potentials, pain, romantic couples

## Abstract

Pain is modulated by social context. Recent neuroimaging studies have shown that romantic partners can provide a potent form of social support during pain. However, such studies have only focused on passive support, finding a relatively late-onset modulation of pain-related neural processing. In this study, we examined for the first time dynamic touch by one’s romantic partner as an active form of social support. Specifically, 32 couples provided social, active, affective (*vs* active but neutral) touch according to the properties of a specific C-tactile afferent pathway to their romantic partners, who then received laser-induced pain. We measured subjective pain ratings and early N1 and later N2-P2 laser-evoked potentials (LEPs) to noxious stimulation, as well as individual differences in adult attachment style. We found that affective touch from one’s partner reduces subjective pain ratings and similarly attenuates LEPs both at earlier (N1) and later (N2-P2) stages of cortical processing. Adult attachment style did not affect LEPs, but attachment anxiety had a moderating role on pain ratings. This is the first study to show early neural modulation of pain by active, partner touch, and we discuss these findings in relation to the affective and social modulation of sensory salience.

## Introduction

Social bonding and support is important for human well-being (Berscheid, 2003; Ditzen and Heinrichs, 2014; Uchino, 2006). Close social bonds, or attachment relationships, have long been suggested to serve safety and distress-alleviating functions (Bowlby, 1969; Mikulincer *et al.*, 2003). Interestingly, evidence from non-human mammals further suggests that it is not the mere presence of conspecifics but rather certain active behaviours (e.g. tactile contact, grooming, licking by conspecifics) that are important for affective regulation (Nelson and Panksepp, 1998). Accordingly, recent proposals suggest that mammals, including humans, have adapted to the presence and active care of other conspecifics, so that our ability to form and regulate emotions (Atzil and Barrett, 2017) and our sense of selfhood (Fotopoulou and Tsakiris, 2017) are constituted on the basis of early social interactions. Thus, according to such theories, social proximity and active social support may constitute the default assumption of the human brain (i.e. social baseline theory; Beckes and Coan, 2011; Coan, 2011) or inherited ‘priors’ in predictive coding accounts (Decety and Fotopoulou, 2015). An ensuing prediction of such theories is that individuals employ fewer higher-order, self-regulatory psychological and neural processes when faced with threats in socially supportive contexts than when alone (Coan, 2011; Eisenberger *et al.*, 2007). Indeed, functional neuroimaging studies have shown an attenuation of neural responses typically implicated in affective regulation (e.g. dorsolateral prefrontal cortex, anterior insula), when social support (e.g. hand-holding by a romantic partner *vs* a stranger) is provided in the face of physical threat (Coan *et al.*, 2006), including pain (Eisenberger *et al.*, 2011; Krahé *et al.*, 2015).

However, the explanatory potential of these neuroimaging studies is restricted in two important ways that we aim to address in this study. The first restriction is that such studies have mostly focused on passive support of one’s partner *vs* control conditions of absence of such support, e.g. presence *vs* absence (Krahé *et al.*, 2015), static hand-holding *vs* no or stranger hand-holding (Coan *et al.*, 2006; Goldstein *et al.*, 2018), viewing pictures of one’s partner *vs* a stranger (Eisenberger *et al.*, 2011). In contrast, there are no neuroscientific studies on active forms of social support from one’s partner, even though in behavioural studies passive and active support have been found to have opposite psychological effects on pain (Krahé and Fotopoulou, 2018; Krahé *et al.*, 2013). Moreover, comparisons between supportive *vs* non-supportive actions (i.e. active support) of the same support provider have greater experimental control and hence explanatory power than many of the manipulations of the above studies, given that they are not subject to confounds such as social distraction, comfort and familiarity. Accordingly, in this study we aimed to examine for the first time the effects of different forms of tactile active social support by one’s romantic partner on pain.

Although there are many ways to provide active support, recent experimental studies (Kirsch *et al.*, 2017; von Mohr *et al.*, 2017) and corresponding theoretical reviews (Fotopoulou and Tsakiris, 2017; Morrison, 2016) suggest that a particular type of dynamic touch may be a particularly effective and salient embodied form of communicating active, social support. Specifically, slow (at 1–10 cm/s velocities), light-pressure (≈0.4 N), dynamic (moving along the skin) touch has been shown to communicate social support (Kirsch *et al.*, 2017) and reduce social pain (von Mohr *et al.*, 2017) in comparison to faster, but otherwise identical, active touch. Importantly, it seems that there may be a dedicated neurophysiological system, the C-tactile (CT) system, coding this particular type of affective touch (Croy *et al.*, 2016; Löken *et al.*, 2009; Olausson *et al.*, 2002; Olausson *et al.*, 2008; Triscoli *et al.*, 2013).

Importantly, this type of touch allows us to address the second major restriction of the existing neuroimaging studies on partner support during pain. Namely, simultaneous manipulations of social touch (e.g. hand-holding) and pain as in previous studies (e.g. Goldstein *et al.*, 2018; see also Coan *et al.*, 2006), does not allow precise inferences about the mechanisms of pain modulation, given the following: (i) the existing, consciously known meaning of hand-holding (i.e. it is not clear whether the observed pain modulation is the outcome of the feeling of being touched or the knowledge about the meaning of handholding), (ii) the well-known analgesic effects of touch on pain (Liljencrantz *et al.*, 2017; Mancini *et al.*, 2015) and (iii) the fact that these studies cannot control for skin-to-skin touch parameters (e.g. pressure of handholding, movement, sweating, temperature) or distraction effects. In contrast, one could control for most of these confounds and their interactions by comparing slow touch, that is known to be mediated by the CT-system and is typically perceived as pleasant, with faster but otherwise identical touch, that is known to not activate the CT system optimally and is typically judged to feel ‘neutral’. Specifically, given that slow, CT-optimal touch can specifically and without prior knowledge signal positive emotions and social support (Kirsch *et al.*, 2017; von Mohr *et al.*, 2017), this manipulation can be done off-line, i.e. not simultaneously with, but before the noxious stimulation, to implicitly signal a socially supportive context to the individual about to receive pain.

Indeed, a recent laser-evoked potential (LEP) study found that individual differences in adult attachment style (i.e, individual differences in the perception of social relationships themselves) determine how a stranger’s slow CT-optimal affective touch (*vs* faster and rated as emotionally neutral but otherwise identical touch), applied before noxious stimulation, affects early responses to noxious stimuli, namely the N1 component (Krahé *et al.*, 2016). While there was no main effect of affective touch on pain in this study and late, cortical responses to pain were not reliably affected (i.e. there was no modulation of the P2 and the N2 was only modulated by an interaction between attachment anxiety and avoidance dimensions), it remains possible that slow, affective touch provided by a romantic partner, where social trust and attachment is already established, might also impact higher-order pain regulation, as captured by later LEP components, i.e. the N2-P2 complex. A romantic partner’s slow affective touch can be more powerful as affective touch is central to intimate, romantic relationships (Suvilehto *et al.*, 2015), and the regulatory role of touch seems to be mediated by psychological intimacy (Debrot *et al.*, 2013).

In sum, the present study goes beyond previous research to investigate the effects of a form of active social support, namely affective touch, on subjective and neural responses to pain in the context of a romantic relationship. Healthy women received slow, CT-optimal touch by their partners *vs* faster, CT non-optimal touch, followed by laser-evoked noxious stimulation, without any other communication between partners. We measured self-reported pain as well as deflections in the ongoing electroencephalogram (EEG) time-locked to transient noxious radiant heat stimulation, namely the N1 and N2-P2 components (Plaghki and Mouraux, 2005), that can tease apart different stages of pain processing: the N1 consists of an early deflection peaking around 160 ms post-stimulus onset and is thought to reflect early sensory (nociceptive) processing preceding conscious awareness (Lee *et al.*, 2009; Valentini *et al.*, 2012), whereas the N2-P2 comprises a later biphasic complex peaking around 200–350 ms post-stimulus onset and is considered to reflect the salience associated with a conscious experience of pain (Lee *et al.*, 2009; Mouraux and Iannetti, 2009). Using these methods, we sought to test two main hypotheses. First, we hypothesised that slow *vs* fast touch would attenuate subjective pain ratings and LEPs reflecting both early and later stages of pain processing, namely the N1 and N2-P2 local peak amplitudes. Second, we expected such effects to be moderated by individual differences in adult attachment style as in previous studies on social support and pain (Hurter *et al.*, 2014; Krahé *et al.*, 2015, 2016; Sambo *et al.*, 2010), in that affective touch should have the largest effect in individuals with higher attachment anxiety (who fear of rejection and seek clear signals of support) and the smallest effect in individuals with higher attachment avoidance (who prefer to cope with threat alone).

## Method

### Participants

Thirty-two couples in a romantic relationship were recruited. We experimentally induced pain in the women (henceforth ‘participants’), while their partners delivered the (slow affective, fast neutral) touch. Participants were included if they were right-handed and had been in their current relationship for over a year. The mean age of participants and their partners was *M =* 24.53 (*s.d.*  = 3.78) and *M =* 26.31 (*s.d.* = 4.65), respectively. See Supplementary Material for other inclusion criteria and sample characteristics. The University College London Research Ethics Committee approved this study, and the experiment was conducted in accordance with the Declaration of Helsinki.

### Design

Our within-subjects design comprised two experimental conditions: slow touch (3 cm/s; affective CT-optimal touch) and fast touch (18 cm/s; neutral, non-CT optimal) administered by the partner—with the order of these conditions counterbalanced across participants. Outcome measures were subjective pain ratings and N1, N2 and P2 local peak amplitudes. The moderating effect of adult attachment style was examined using a questionnaire that measures the degree of attachment anxiety and avoidance that individuals may experience in close, adult romantic relationships [Experiences in Close Relationships-Revised (ECR-R); Fraley *et al.*, 2000].

### Materials and measures

#### Tactile stimulation

Two skin areas (9 cm long × 4 cm wide) were marked on the participant’s right forearm (i.e. stimulation sites). The partner administered the touch to the participant using a cosmetic make-up brush (Natural Hair blush brush, No. 7, Boots, UK). The partner was trained to administer each touch condition by watching a 4 min video and then practicing the touch on the second experimenter outside the testing room. In each touch condition, the stroking was administered in four 45 s mini-blocks in an elbow-to-wrist direction (Essick *et al.*, 2010; Krahé *et al.*, 2016) at slow (3 cm/s, 1 stroke) or fast (18 cm/s, 6 strokes) velocities. The velocities of the slow and fast touch were chosen as they have been shown to be optimal and non-optimal, respectively, for targeting CT afferents (Löken *et al.*, 2009; Gentsch *et al.*, 2015). Further, these same velocities have also been validated in our previous studies and have revealed statistically significant differences in their effects on social and physical pain (Krahé *et al.*, 2016; von Mohr *et al.*, 2017) and the communication of social support (Kirsch *et al.*, 2017). The 3 s stroking was alternated with 3 s pauses. Stimulation sites were also alternated between trials to avoid CT habituation.

#### Nociceptive stimulation and subjective pain report

We used an infrared CO_2_ laser stimulation device with a wavelength of 10.6 μm (SIFEC, Ferrières, Belgium) to deliver noxious radiant heat stimulation. The laser stimulus (80 ms duration, spot diameter of 6 mm) was applied to the dorsum of participant’s left hand, changing the stimulation site between consecutive applications. Using an ascending-descending-ascending staircase, we identified each participant’s Aδ threshold for ‘pinprick pain’ (i.e. the lowest skin temperature that elicited a report of ‘pinprick sensation’, which is linked to Aδ fibres; Lee *et al.*, 2009). The pain threshold (*M* = 47.65°C, *s.d.* = 2.35) was used to set a mild-to-moderate (but always tolerable) sharp pinprick sensation (3°C above threshold, experimental trials) and no pinprick sensation (2°C below threshold, distractor trials) (See Supplementary Material for details).

Each block consisted of 60 laser stimuli (40 experimental stimuli and 20 distractor stimuli), presented in pseudorandom order with an interstimulus interval of 10–15 s. Participant’s self-reported pain intensity was recorded using a numeric keyboard on an 11-point scale ranging from 0 (no pinprick sensation) to 10 (extremely painful pinprick sensation). Mean pain ratings for the experimental stimuli in each block (across the four mini-blocks for the touch conditions) were used as the measure of subjective pain report. Data exclusion due to technical issues resulted in a final sample size of 31 (*N* = 31).

#### EEG recording and LEP analyses

The study was carried out using a BioSemi ActiveTwo EEG system (http://www.biosemi.com; Biosemi, Amsterdam, The Netherlands) with a 64-electrode cap. A BioSemi analog input box connected to the analog output of the laser stimulation device was used to record the online measurement of skin temperature at target laser stimulation site in register with the EEG recording across the experimental task. The electrooculography was monitored with a total of four electrodes located at the outer canthi of both eyes as well as above and below the right eye. The sampling rate during recording was 1024 Hz.

EEG data were processed and prepared for statistical analysis using EEGLAB/ERPLAB toolboxes for MATLAB (R2015b) (see Supplementary Material for EEG data pre-processing details). Average waveforms per condition were computed (experimental trials only). For each waveform, the peak amplitude of the N1, N2 and P2 were measured as follows: the N1 was measured at the central electrode contralateral to the stimulated side (C6), referenced to Fz (Krahé *et al.*, 2015, 2016). It was defined as the most negative deflection following stimulus onset and preceding the N2 wave (Lee *et al.*, 2009). The N2 and P2 were measured at the vertex (Cz) referenced to the average of P9 and P10 (electrodes close to the mastoids; Luck, 2014). The N2 and P2 were defined as the most negative and positive deflection, respectively, after stimulus onset (Lee *et al.*, 2009). In accordance with prior literature reporting that the earliest neural activity associated with laser stimulation occurs after 120 ms (Valentini *et al.*, 2012), no deflection occurring before 120 ms after stimulus onset was selected as the peak (Krahé *et al.*, 2015). Data exclusion due to technical issues resulted in a final sample size of 29 (*N* = 29) for N1 and 29 (*N* = 29) for N2 and P2 analyses (see Supplementary Material).

#### Adult attachment style

We employed the ECR-R (Fraley *et al.*, 2000) to measure adult attachment style. This questionnaire is designed to measure individual differences with respect to the extent to which individuals are insecure about the responsiveness and availability of their romantic partners (i.e, attachment anxiety) and the extent to which individuals are uncomfortable with being close and depending on their romantic partners (i.e. attachment avoidance). The ECR-R consists of 36 items on a 7-point scale and yields continuous scores on attachment anxiety and attachment avoidance dimensions, with higher scores denoting greater attachment anxiety and avoidance, respectively. The ECR-R is a well-validated measure (Ravitz *et al.*, 2010). In the present sample, Cronbach’s alpha was α = 0.86 for attachment anxiety and α = 0.89 for attachment avoidance.

#### Manipulation checks

As a manipulation check, participants were asked to rate retrospectively how comfortable they had felt with the touch velocities delivered by their partner in each condition. These reports were given on a scale ranging from −3 (not at all comfortable) to 3 (extremely comfortable). We also collected pleasantness ratings of the touch to make sure participants perceived slow touch as more pleasant than fast touch. Here, participants received 12 randomized trials of 3 s stroking at slow (3 cm/s) and fast (18 cm/s) velocities, the same touch velocities as in the main task. Using a scale ranging from 0 (not at all pleasant) to 100 (extremely pleasant), participants were asked to rate the pleasantness of the touch after each trial. Slow and fast touch ratings were averaged separately for each participant.

**Fig. 1:**
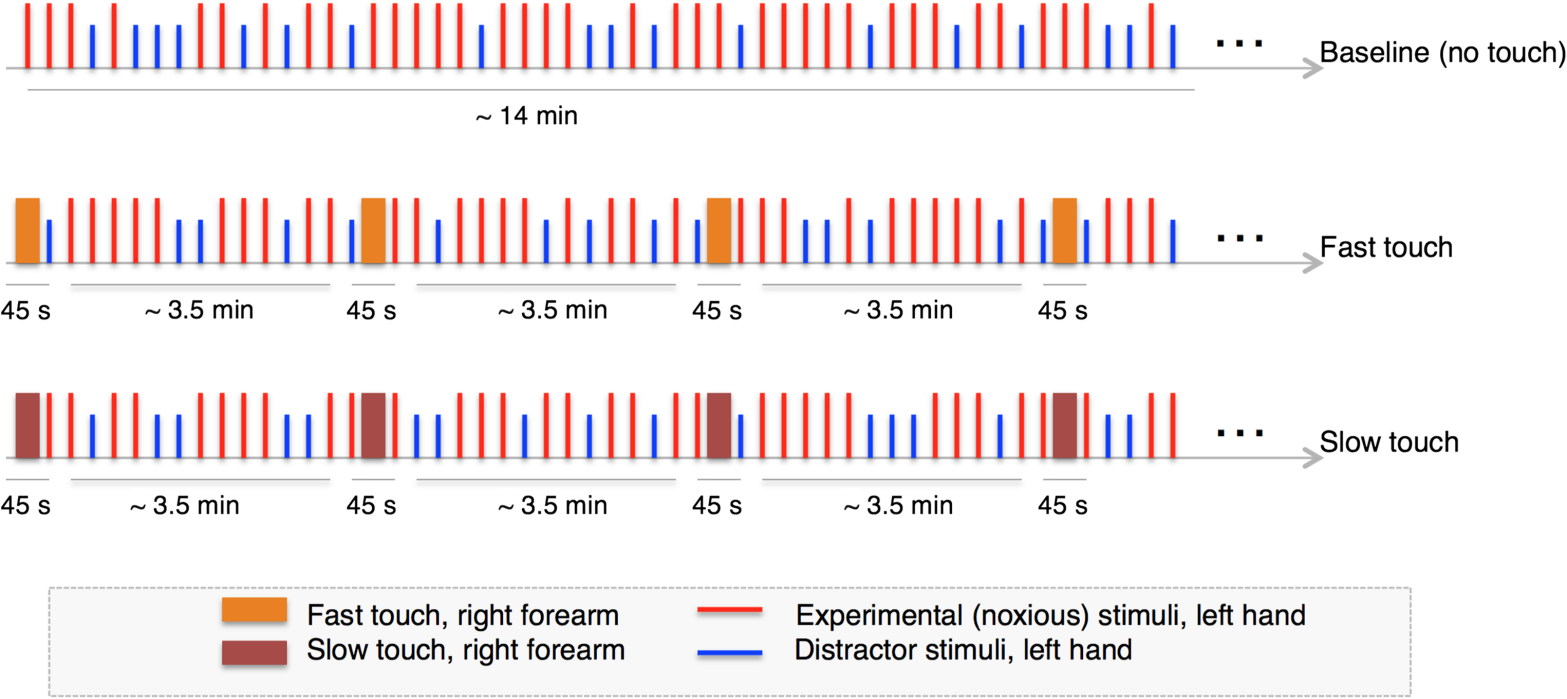
Experimental design. Our experimental design for the main task included a baseline (no touch) nociceptive block followed by a fast touch or a slow touch block. The order of the touch (fast or slow) blocks was counterbalanced across participants. The laser stimuli (experimental and distractor trials) were presented in pseudorandom order with an interstimulus interval of 10–15 s.

**Table 1:** Mean (s.d.) for pain-related outcome measures

	Baseline (no touch)	Slow touch	Fast touch
N1 local peak amplitude (μV)	−5.33 (3.27)	−3.50 (2.23)	−4.20 (2.60)
N2 local peak amplitude (μV)	−10.90 (7.09)	−5.92 (4.61)	−7.52 (5.08)
P2 local peak amplitude (μV)	16.02 (10.14)	9.65 (7.48)	11.81 (6.42)
Pain ratings	4.06 (1.73)	3.01 (1.84)	3.58 (1.82)

### Procedure

Upon obtaining written informed consent, each participant’s pinprick pain threshold was determined, and the experimental and distractor laser intensities to be used in the main task were set based on this threshold. The experiment consisted of three laser blocks. Participants did not see or speak with their partners during any of the blocks, and they were prevented from seeing the stimulated skin areas through the use of a black box placed around the stimulated arms.

In the first block, we recorded participant’s EEG while administering a baseline nociceptive stimulation block (no touch). In the two other blocks, participants received one of the two stroking velocity conditions (slow or fast touch) from their partner, followed by noxious stimuli (with the order of the stroking velocity conditions counterbalanced across participants). Each of these touch blocks was divided into four mini-blocks, alternating tactile stimulation with noxious stimulation (tactile and noxious stimulation were administered in spatial and temporal incongruence and asynchrony in order to avoid concurrent multisensory effects; see Figure 1 for a schematic of the experimental design). Between each block, there was a 7 min break with Sudoku and/or crossword puzzle to minimise carryover effects; in the meantime, the partner was trained with the other touch velocity she/he was about to deliver to the participant. EEG was recorded throughout the periods of laser stimulation following slow and fast touch conditions. At the end of the study visit (∼120 mins), participants were asked retrospectively to rate how comfortable they felt with the touch provided by their partner in each condition.

**Table 2:** Slow *vs* fast touch: multilevel modelling results for all outcome measures

Effect	Dependent variable	b	*SE*	*P*-value	Confidence intervals	
					Lower	Upper
**Slow touch *vs* fast touch**	**N1**	**−0.97**	**0.46**	**0.036**	**−1.88**	**−0.06**
	**N2**	**−2.06**	**0.76**	**0.007**	**−3.54**	**−0.57**
	**P2**	**2.85**	**0.89**	**0.001**	**1.12**	**4.59**
	**Pain ratings**	**0.62**	**0.13**	**<0.001**	**−0.37**	**0.86**
Attachment anxiety	N1	−0.21	0.54	0.691	−1.28	0.85
	N2	−1.35	0.86	0.116	−3.03	0.33
	P2	1.61	1.18	0.17	−0.69	3.92
	Pain ratings	0.01	0.20	0.97	−0.39	0.41
Attachment avoidance	N1	0.05	0.60	0.939	−1.13	1.22
	N2	−0.67	0.96	0.488	−2.56	1.22
	P2	−1.45	1.32	0.270	−4.03	1.13
	Pain ratings	−0.06	0.22	0.787	−0.49	0.37
**Attachment anxiety × attachment avoidance**	N1	−0.77	0.81	0.343	−2.36	0.82
	**N2**	**−2.70**	**1.22**	**0.028**	**−5.11**	**−0.29**
	P2	0.97	1.68	0.564	−2.33	4.27
	Pain ratings	−0.02	0.29	0.945	−0.59	0.55
**Touch condition × attachment anxiety**	N1	1.17	0.64	0.068	−0.09	2.43
	N2	1.26	1.04	0.229	−0.79	3.31
	P2	−2.01	1.23	0.101	−4.41	0.393
	**Pain ratings**	**−0.41**	**0.18**	**0.023**	**−0.76**	**−0.05**
Touch condition × attachment avoidance	N1	0.28	0.74	0.709	−1.17	1.72
	N2	2.19	1.17	0.061	−0.10	4.50
	P2	1.78	1.37	0.195	−0.91	4.48
	Pain ratings	−0.15	0.19	0.442	−0.22	0.51
Touch condition × attachment avoidance × attachment anxiety	N1	0.19	1.05	0.854	−1.87	2.26
	N2	1.31	1.50	0.384	−1.64	4.25
	P2	−2.22	1.76	0.207	−5.67	1.23
	Pain ratings	−0.26	0.26	0.305	−0.76	0.24

Note. Significant main effects and interactions are highlighted in bold. Same pattern of results were observed when controlling for relationship quality (see Supplementary Material, Table S1). While the interaction between attachment anxiety and attachment avoidance was statistically significant, follow-up tests were non-significant/trend level (see Supplementary Material, Figure S1). Baseline pain as a covariate was statistically significant across all pain outcomes, *P* < 0.05.

To avoid biasing the results of the main pain task, participants returned for a second visit, between 3 and 5 days after the first visit, in which they completed the adult attachment style questionnaire (ECR-R) and provided pleasantness ratings for slow and fast touch. Participants were paid £50 for their (and their partner’s) time and were fully debriefed at the end of the second visit.

### Plan of statistical analyses

All statistical analyses were conducted in STATA (Version 14). As repeated measures (Level 1) were nested within individuals (Level 2), multilevel modelling was implemented. For each outcome variable (pain ratings and N1, N2 and P2 local peak amplitude), we specified multilevel models with touch condition (slow touch/fast touch) as a dummy-coded categorical predictor, attachment avoidance and attachment anxiety as continuous predictors, and included all interaction terms. We controlled for pain baseline differences by including them as covariates (see also Supplementary Material, Table S1 for analyses controlling for relationship quality in our models). All continuous predictors were mean-centred in order to avoid multicollinearity issues (Tabachnick and Fidell, 2007). Given that multilevel models address the multiple comparisons problem and yield more efficient estimates (Gelman *et al.*, 2012), we did not correct for multiple comparisons any further in our model. Significant interactions were followed up by examining differences between conditions at low (−1 s.d.), moderate (mean) and high (+1 SD) continuous attachment style scores (see Aiken and West, 1991 for testing and interpreting interactions on continuous variables).

## Results

### Descriptive statistics and manipulation checks

Mean adult attachment scores were *M* = 2.50 (*s.d.* = 0.75) for attachment anxiety and *M* = 2.55 (*s.d.* = 0.69) for attachment avoidance (see Supplementary Material, Table S2 for comparisons with the general population). Attachment anxiety and avoidance dimensions were correlated at *r* = 0.35, *P* < 0.05. On average, participants reported good relationship quality/adjustment (*M =* 25.84, *s.d.* = 3.18) as measured by the seven-item Dyadic Adjustment Scale (Sharpley and Rogers, 1984). Relationship quality/adjustment did not correlate with attachment anxiety or avoidance dimensions (see Supplementary Material). Table 1 presents descriptive statistics for pain ratings and associated neural responses (N1, N2 and P2 local peak amplitudes).

As expected, participants reported feeling more comfortable with slow touch (*M* = 2.38, *s.d.* = 1.16), as compared to fast touch (*M* = 1.41, *s.d.* = 1.74) from their partner, *t*(31) = 3.67, *P* = 0.001. Participants also reported higher pleasantness in response to slow touch (*M* = 72.15, *s.d.* = 13.48), as compared to fast touch (*M* = 54.72, *s.d.* = 14.16), *t(*31) = −5.89, *P* < 0.001. Thus, our manipulations were successful in terms of perceived pleasantness and comfort of the touch.

### Do slow, affective touch and adult attachment style—alone and in interaction—modulate pain and associated neural responses?

#### Main effects

**Fig. 2:**
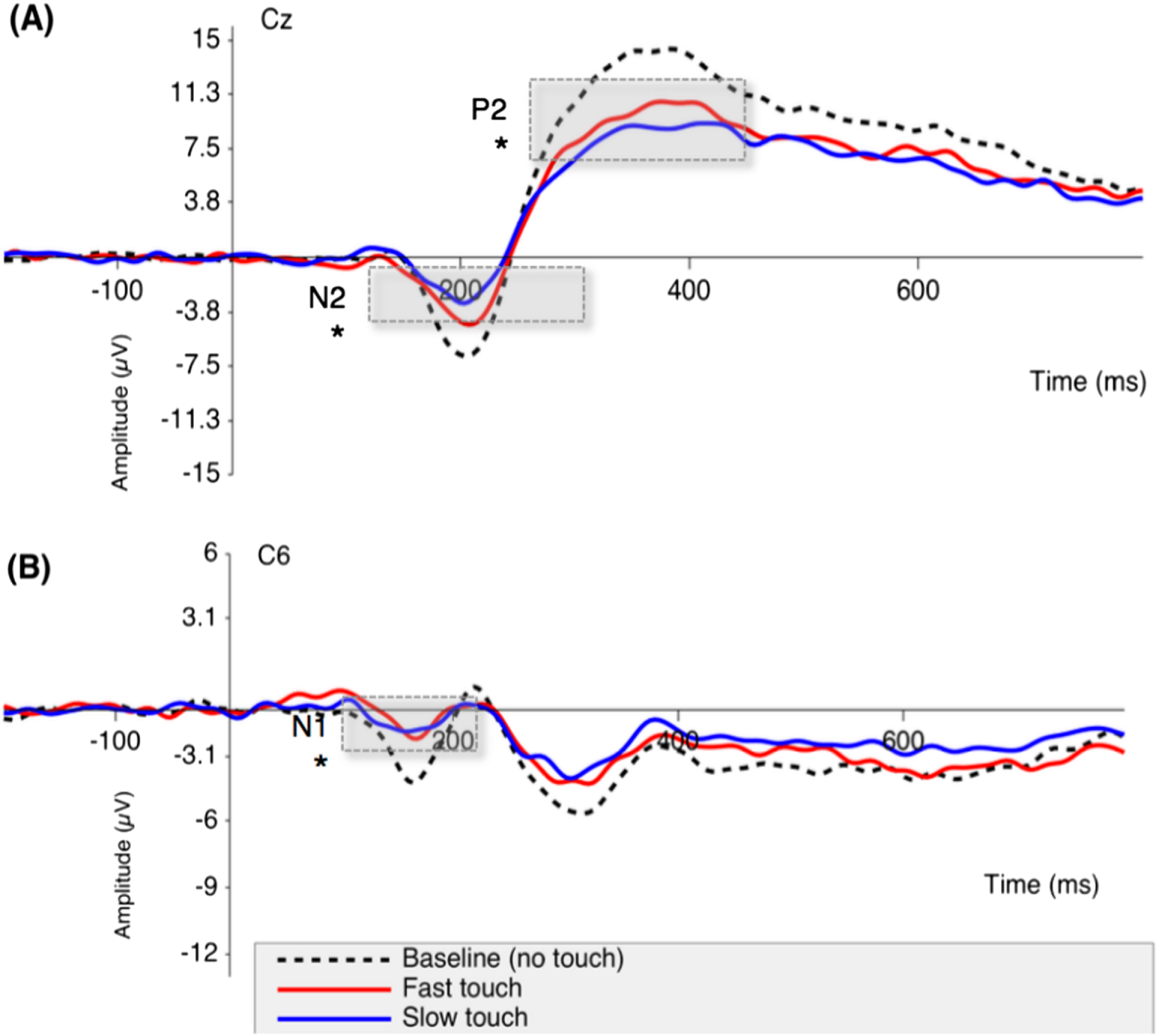
(A) Effect of touch condition on the N2-P2 waveform measured at the vertex (Cz). (B) Effect of touch condition on the N1 waveform measured at the contralateral side of stimulation (C6). N1, N2 and P2 local peak amplitude was significantly smaller in the slow touch compared to the fast touch condition, as denoted by asterisks. Baseline pain (no touch) as a covariate was statistically significant across the N1, N2 and P2 local peak amplitude.

Full model results are presented in Table 2. Supporting our first hypothesis, a significant main effect of touch condition was found on the subjective pain ratings, N1, N2 and P2 local peak amplitudes. All our effects were in the same direction: Regarding the pain ratings, participants reported less pain in the slow touch (*M* = 3.01, *SE*  = 0.13) compared to the fast touch (*M* = 3.58, *SE* = 0.13) condition (see Table 2). With respect to the neural responses associated with pain, the N1 local peak amplitude was significantly smaller in the slow touch (*M* = −3.48 μV, *SE* = 0.37) compared to the fast touch (*M* = −4.28 μV, *SE* = 0.39) condition; the N2 local peak amplitude was significantly smaller in the slow touch *(M* = −5.92 μV, SE = 0.59) compared to the fast touch (*M* = −7.65 μV, *SE* = 0.59) condition, and the P2 local peak amplitude was significantly smaller in the slow touch (*M* = 9.65 μV, *SE* = 0.80) compared to the fast touch (*M* = 12.11 μV, *SE* = 0.80) condition. No other main effects were significant (see Table 2). Together, these results suggest that pain report and associated neural responses were attenuated in response to slow, affective touch relative to fast, neutral touch (see Figure 2 for N1, N2-P2 waveforms).

#### Touch condition in interaction with adult attachment style

**Fig. 3:**
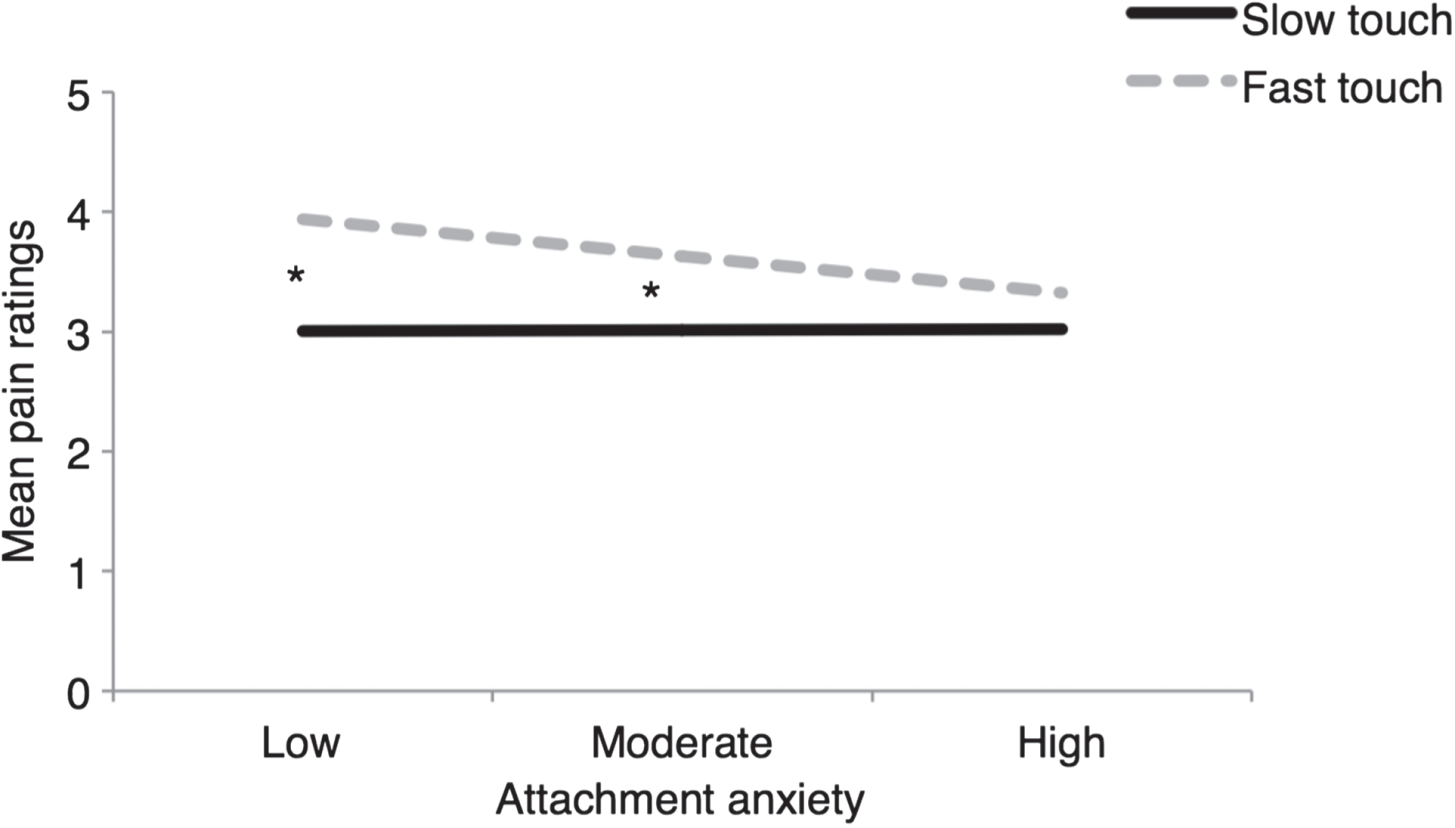
Touch condition by attachment anxiety effects for pain ratings. Statistically significant differences are marked by asterisk, *P* < 0.05. Participant’s self-reported pain intensity was recorded on an 11-point scale ranging from 0 (no pinprick sensation) to 10 (extremely painful pinprick sensation).

Partially supporting our second hypothesis, we found a significant touch condition by attachment anxiety interaction on pain ratings, *b* = −0.41, SE = 0.18, *P* = 0.023, but not on the neurophysiological outcome measures. Follow-up tests on the pain ratings showed that the difference between slow and fast touch conditions was significant for low (*b* = −0.93, SE = 0.20, *P* < 0.001) and moderate attachment anxiety (*b* = −0.62, SE = 0.13, *P* < 0.001), but not for high attachment anxiety (*b* = −0.31, SE = 0.17, *P* = 0.074); see Figure 3. Thus, the higher the attachment anxiety, the smaller was the difference between slow and fast touch on pain ratings, i.e. at high levels of attachment anxiety, slow and fast touch did not differ in terms of their effects on pain ratings. There was no significant two-way interaction between attachment dimensions and no three-way interaction of touch condition, attachment anxiety and attachment avoidance on the pain ratings, indicating that these results were driven by the attachment anxiety dimension. Contrary to our second hypothesis, the interaction between touch condition and attachment avoidance was non-significant for all outcome measures.

## Discussion

While passive social support from one’s romantic partner can have pain-attenuating effects and corresponding modulation of neural responses (Eisenberger *et al.*, 2011; Goldstein *et al.*, 2018; Krahé *et al.*, 2015), little is known about the effects of active partner support on pain. Here, we investigated the effects of partner CT-optimal touch on pain, given the experimentally established role of this type of touch in the communication of positive emotions and social support (Kirsch *et al.*, 2017; von Mohr *et al.*, 2017). We found that slow, affective *vs* fast, neutral touch from one’s partner reduces subjective pain ratings and similarly attenuates LEPs both at earlier (N1) and later (N2-P2) stages of cortical processing. Contrary to our second hypothesis, adult attachment style did not affect LEPs as in other social contexts (Krahe *et al.*, 2016), but one facet of adult attachment style, namely attachment anxiety, had a moderating role on self-reported pain. These findings are discussed in more detail below.

Regarding our first hypothesis about the role of active, affective touch on pain reduction, we found such effects on pain report and LEPs reflecting both early and later stages of cortical nociceptive processing, namely the N1 and N2-P2 local peak amplitude. Our findings on the N2-P2 complex, which has been linked to activity in areas such as the anterior insula and anterior cingulate cortex (Garcia-Larrea *et al.*, 2003) and to late, conscious aspects of noxious processing (Lee *et al.*, 2009; Mouraux and Iannetti, 2009), are consistent with previous neuroimaging studies on passive social support (e.g. Eisenberger *et al.*, 2011; Krahé *et al.*, 2015), which found similar downregulation of brain areas supporting conscious aspects of noxious processing, such as the anterior insula and dorsal anterior cingulate cortex (Eisenberger *et al.*, 2011). However, contrary to these studies, our N1 findings indicate that the effects of active, affective touch may begin at earlier stages of cortical nociceptive processing. Even though the N1 wave represents an early stage of sensory processing more directly related to ascending nociceptive input (Lee *et al.*, 2009; Valentini *et al.*, 2012), such cortical encoding is already ‘late’ in the grand scheme of noxious encoding. The N1 has been linked to activation in the operculoinsular and primary somatosensory cortex (Garcia-Larrea *et al.*, 2003; Valentini *et al.*, 2012), and such initial cortical coding of noxious afferent inputs is considered an essential, yet distinct stage of signal processing, different from later stages that are associated with the conscious aspects of pain and its affective regulation.

It is unlikely that the LEP downregulation we observed is based on potential interactions between nociceptive and CT pathways at the spinal cord level (Liljencrantz *et al.*, 2017; Mancini *et al.*, 2015), as the tactile and noxious stimulation in our study were delivered at different times and in different body locations. Instead, given that LEPs have been recently proposed to detect environmental threat to the body in response to sensory salient events (Legrain *et al.*, 2011; Mouraux and Iannetti, 2009), we speculate that affective touch by one’s romantic partner when applied before noxious stimulation may reduce the sensory salience of impending noxious stimulation. Salience has various definitions. Here, we use the term to describe the importance of a stimulus (its weighting in relation to other factors) for indicating potential or actual threat to the body (Legrain *et al.*, 2011) and for inducing related responses (Garcia-Larrea and Peyron, 2013). Conceptualising the LEP-related brain activity as being part of a ‘salience network’ (Legrain *et al.*, 2011), we have recently proposed that activity in this salience network may also be modulated by information regarding contextual factors from the (social) environment (Krahé and Fotopoulou, 2018; Krahé *et al.*, 2013; von Mohr and Fotopoulou, 2018; see also Atlas and Wager, 2012; Büchel *et al.*, 2014 for the role of expectations and active inference). Although the precise neurophysiological mechanisms of the effects of active, affective touch on pain will need to be studied in future studies, we discuss below four possible explanations of how the sensory salience of noxious stimulation may have been moderated in this study.

First, it is well known that pain can be modulated by distraction. While our study controls various facets of social distraction better than previous studies (see Introduction), similarly to other pain modulation studies, we cannot exclude with absolute certainty that our two touch conditions did not have some difference in their general attentional demands. For example, neutral touch delivered at fast speeds might demand greater attention than slow, affective touch (see also Davidovic *et al.*, 2017 for an attenuation of the Default Mode Network in response to non-affective touch). However, we think this is unlikely because if fast touch was, in fact, more attention grabbing and thus distracting, we would expect fast *vs* slow touch to attenuate pain. Instead, our findings show the opposite pattern and as discussed below, it is possible that the mechanism by which affective touch can selectively modulate pain may relate to its salience.

Second, given the many factors that can influence pain when comparing across different socially supporting contexts, e.g. individual factors, habituation, distraction, mood, social presence, familiarity and many more, our aim was to compare directly two specific and well-controlled types of touch, namely slow and fast stroking on the forearm by the same, familiar person at different times and in different body locations than the noxious stimulation. To address this aim optimally, we have elected to use a within-subjects design, measuring individual pain measures before any manipulation, and subsequently counterbalancing order between our two critical conditions. While this design is optimal for assessing whether pain responses differ between the two critical conditions, it does not allow us to disentangle the potential general effects of touch on pain (beyond the critical manipulation of stroking speed) and the general effects of condition order on pain. In other terms, baseline always precedes the two touch conditions, and hence any general touch effects on pain could be due either to the touch or the fact that the touch conditions come always after the baseline condition and hence may be subject to habituation effects. However, we can say that there is an effect of slow, affective touch *vs* fast, neutral touch on pain over and above any order effects, as their order was counterbalanced across conditions. It is still possible, however, that fast, neutral touch may be increasing pain in comparison to slow touch rather than the other way around. As we previously said, we cannot disentangle the role of habituation from the role of general touch in our studies, and hence it is possible that fast, neutral touch was associated with less habituation than slow touch in our study. However, we also note that neutral touch has long being known to reduce rather than increase pain in previous studies (e.g. see Mancini *et al.*, 2015 for recent study) and future studies could thus include further speeds to account for the direction of the observed effects.

Third, given that CT firing correlates with perceived pleasantness in response to dynamic stroking (Löken *et al.*, 2009), with our own findings also suggesting increased perceived pleasantness in response to slow (*vs* fast) touch, it is possible that affective touch may reduce the sensory salience of impending noxious stimulation in a similar way as positive mood-related manipulations (e.g. positive/pleasant pictures, music and odours, see Villemure and Bushnell, 2002, for a review). Given the importance for quick and unbiased experimental succession between touch and pain, we did not collect mood ratings in this experiment. Instead, we merely examined, as an off-line manipulation check, the perceived sensory pleasantness of slow and fast touch. Interestingly, the degree to which participants perceived slow and fast touch to be pleasant was not related to pain modulation in the corresponding conditions (similar to Krahé *et al.*, 2016; see Supplementary Material, Table S3). Thus, future studies should include specific online measures of mood to further explore this hypothesis.

Fourth, CT-optimal touch is a particularly effective form of communicating embodied (non-verbal) social support (Kirsch *et al.*, 2017; von Mohr *et al.*, 2017). Specifically, recent evidence on this very modality suggests that this particular kind of slow dynamic touch, but not the faster stroking touch also tested here as a control condition, conveys positive social intentions such as social support even in the absence of any other sensory or social cue (Kirsch *et al.*, 2017). Thus, it is possible that this type of touch attenuates the saliency of impending noxious stimuli by signalling the presence of an active, socially supportive environment. This interpretation is consistent with recent theories on the importance of social interactions for the experience and regulation of emotions, and particularly homeostatic emotions such as pain (Atzil and Barrett, 2017; Fotopoulou and Tsakiris, 2017). According to such theories, the perception of the social environment of pain can affect inferential processes about the perception of these modalities by influencing the weighting of prior expectations about certain sensory signals *vs* the signals themselves in given contexts (Decety and Fotopoulou, 2015; Krahé *et al.*, 2013; von Mohr and Fotopoulou, 2018; similar to how non-social expectations influence pain, e.g. Atlas and Wager, 2012; Geuter et al., 2017). Accordingly, affective touch prior to a noxious stimulus may modulate pain by changing beliefs about how threatening a noxious stimulus is in a supportive social context. Future studies should thus also take direct measure of perceived social support and examine whether the latter possibility or more general positive mood effects best explain the effects of CT-optimal touch on pain. In addition, future studies could elucidate whether the effects of slow, affective touch on pain are specific to the CT system. For example, could slow touch to glabrous skin, that does not possess CT fibres (McGlone *et al.*, 2014), lead to similar effects in romantic couples?

Turning now to our second study hypothesis about the potential moderating role of adult attachment style based on the observation of such effects in different social contexts (e.g. Krahé *et al.*, 2016), we found such an effect only on subjective pain ratings and only in relation to adult attachment anxiety. Specifically, the higher the attachment anxiety, the smaller the effects of slow *vs* fast touch on self-reported pain. Given that anxious attachment is associated with craving closeness and reassurance from others (Hazan and Shaver, 1987), we hypothesise that any kind of physical contact, in this case slow or faster touch from one’s partner, is enough to ease attachment anxiety, signal closeness and hence attenuate self-reported pain. The fact that we did not observe any other attachment effects on our pain measures as in earlier work on affective touch between strangers (Krahé *et al.*, 2016), may be explained by the fact that individual differences in attachment may have less of a role to play when there is an existing degree of attachment security between partners. The latter can be assumed in the partners of the current study who were in a relationship for at least 12 months, had good relationship quality (see Supplementary Material) and showed relatively secure attachment in relation to existing norms on the same measure and our previous study (see Supplementary Material, Table S2).

To the best of our knowledge, our study was the first to investigate the effects of active, affective touch on pain in the context of a romantic relationship. A recent LEP study by our lab suggests that there are no main effects of affective touch on pain when a stranger (confederate) administers the touch, but rather these effects depend on attachment style, mostly modulating early stages of cortical processing, namely the N1 component. Specifically, even though the N2 mirrored the effects on the N1, such effects by attachment style (e.g. high anxiety) were observed only in relation to the other attachment dimension (e.g. low avoidance), and other later cortical responses to pain such as the P2 were not affected (Krahé *et al.*, 2016). Thus, here we extend these findings to suggest that this type of active embodied social support can modulate not only early (N1) but also later (N2-P2) stages of cortical processing. Critically, and unlike the first study with confederates, these effects on early and later stages of cortical processing by affective touch were not moderated by adult attachment style, which may well pertain to the social context studied in the present study (i.e. romantic couples). Indeed, a romantic partner’s affective touch can be more powerful as affective touch is central to intimate, romantic relationships (Suvilehto *et al.*, 2015; Croy *et al.*, 2016) and the regulatory role of touch seems to be mediated by psychological intimacy (Debrot *et al.*, 2013).

More generally, while there are many ways to provide active support during pain (e.g. supportive text messages, verbal reassurance, social distraction), the current findings are important given that the only variable manipulated was the velocity of the touch from the romantic partner. Thus, we demonstrate that a simple, yet specific embodied interaction can have pain-attenuating effects without the need for any explicit labelling by words or pictures. Moreover, in comparison to other types of embodied social support, such as for example hand-holding, the tactile interaction studied here was manipulated with a degree of experimental control, at a time different than the noxious stimulation and tested against control conditions that involve the same support provider. Therefore, the problematic comparison between partners and strangers or friends could be avoided and many confounding factors, such as social proximity, familiarity and social desirability can be excluded as potential explanations of our effect.

However, despite these methodological advantages, our study had several limitations. First, the experimental control of the study limits its ecological validity as typically couples will use a much richer embodied and verbal interaction to provide social support. Relatedly, in order to be able to assess the effects of touch on pain, including LEPs, as well as to avoid concurrent multisensory effects, the touch was delivered in advance, and repeated in mini-blocks, of ‘impending’ noxious stimuli. However, it is likely that romantic couples will also use this type of embodied social support as a soothing, consoling touch during or after pain and future studies could explore any differences based on such timescales. Second, while this study examined the pain-attenuating effects of touch delivered at velocities that activate the CT system optimally *vs* velocities of minimal known activation of this system (Löken *et al.*, 2009), the functional role of this system and its particular, neurophysiological contribution to our effects remain to be specified by future studies. Third, we only tested pain in women, while their partners provided support by touch, to control for gender effects associated with the perception of touch (Gazzola *et al.*, 2012; Suvilehto *et al.*, 2015); however, future research is needed to examine whether the present results extend to men. Finally, the sources of the N1 and its functional implications remain debated: most notably, in relation to the precise contribution of the primary somatosensory cortex and operculoinsular cortex (Iannetti *et al.*, 2005; Tarkka and Treede, 1993; Valentini *et al.*, 2012) as well as its implications for perceptual *vs* pre-perceptual pain processing at such early stages.

In sum, we found that active touch administered by the romantic partner at the optimal velocities of the CT system prior to noxious laser stimulation at a different body part attenuated subjective pain ratings and neurophysiological responses to pain, namely the N1, N2 and P2 local peak amplitudes more than touch administered at non-optimal CT velocities. Such effects were moderated only by one facet of adult attachment style (attachment anxiety) and only for subjective ratings of pain rather than the neurophysiological measures. Our effects indicate that the analgesic effects of active affective touch may begin at earlier stages of cortical nociceptive processing, as reflected by the N1 local peak amplitude, and expand to later, conscious aspects of noxious processing, as reflected by the N2-P2 complex and self-reported pain ratings. Given that LEPs have been recently proposed to detect environmental threat to the body in response to sensory salient events (Legrain *et al.*, 2011), we propose that affective touch by one’s romantic partner (when applied before noxious stimulation) may reduce the sensory salience of impending noxious stimulation, due to either its perceived affective or pro-social effects.

## Supplementary Material

Supplementary Data
