# Supplementary material for: The social buffering of pain by affective touch: a laser-evoked potential study in romantic couples
Source: Soc Cogn Affect Neurosci. 2018 Sep 24;13(11):1121–30. doi: 10.1093/scan/nsy085 (PMC6234321; doi:10.1093/scan/nsy085)
Supplement: Supplementary Data [file nsy085_supp.doc]

**Supplementary Material**

**Participants**

**Inclusion criteria.** Participants were included if they were right-handed, 18-30 years old, had been in their current relationship for over a year, had no history of chronic pain, psychiatric or neurological disorder, had a depression severity score <9 (PHQ-9 questionnaire; Kroenke, Spitzer, & Williams, 2001), and had no tattoos or skin irritation/disease on the forearms or hands.

**Sample characteristics.** The mean age of participants and their partners was *M=*24.53 (*SD*=3.78) and *M=*26.31 (*SD*=4.65), respectively, and had been in their current relationship for *M*=34.38 months (*SD*=26.24). Participants indicated they were Asian/Asian British (40.63%), White/Caucasian (31.25%), Hispanic/Latin-American (18.75%) and Multi-racial/other (9.38%). Couples were heterosexual (84.38%) and homosexual (15.62%).

**Pain threshold procedure and forced-choice discrimination task**

Using an ascending-descending-ascending staircase, we identified each participant’s Aδ threshold for ‘pinprick pain’ (i.e., the lowest skin temperature that elicited a report of “pinprick sensation”, which is linked to Aδ fibres; Lee et al., 2009). Beginning at 38°C, the laser temperature increased by steps of 4°C until the participant reported a clear pinprick sensation. Next, the temperature was decreased by steps of 2°C until the participant reported no pinprick sensation. Finally, the temperature was increased by steps of 1°C until the participant reported a pinprick sensation for three consecutive repetitions of the same temperature. The pain threshold (*M*=47.65 °C, *SD*=2.35) was used to set a mild-to-moderate (but always tolerable) sharp pinprick sensation (3°C above threshold, i.e., experimental trials) and no pinprick sensation (2°C below threshold, distractor trials). To ensure that participants were able to discriminate between these two laser intensities (i.e., 3°C above threshold, 2°C below threshold), they performed a forced-choice task in which they received a total of 20 randomised laser stimuli (10 stimuli set at the experimental intensity and 10 at the distractor stimuli intensity) and had to identify whether or not they felt the pinprick. If the percentage of correct trials was higher than 75%, we proceeded to the main experimental task. However, if participants scored less than 75% correct, their laser distractor intensity was decreased by 3°C (instead of 2°C) and we repeated the force-choice task (this was the case for two participants).

**EEG data pre-processing details**

EEG data were processed and prepared for statistical analysis using EEGLAB/ERPLAB toolboxes for MATLAB (R2015b). Triggers for each experimental trial were set offline according to each participant’s individual pinprick threshold by using the continuous skin temperature measurements recorded by the laser stimulation device. Data were then downsampled to 256Hz and digitally filtered (between .4 and 30 Hz) to reduce environmental noise. The EEG signal was then segmented into one-second event-related epochs (200 ms before and 800 ms after stimulus onset) and baseline-corrected. Visual inspection was used to remove bad scalp channels and bad eye channels, and trials with muscle and eye blink artifacts were rejected. Only averaged potentials for experimental trials were analyzed. We measured N1 and N2-P2 local peak amplitudes for individuals with at least 65% experimental trials following artifact rejection (as in^30^). On average, participants had 91.7% (*SD*=7.58%), 89.85% (*SD*=6.48%) and 89.93% (*SD*=8.23%) experimental trials for the pain baseline (no touch), slow touch and fast touch conditions, respectively.

**EEG Data Exclusions**

One participant was excluded because she had fewer than 65% usable experimental trials, i.e., trials without artifacts. Further, two participants were excluded from the N1 and the N2-P2 analyses given that no plausible peak could be identified across any of the experimental conditions. For participants for whom we had average potentials available and had plausible potentials in at least one condition, we estimated the missing data using the maximum likelihood estimation command in the multilevel modelling analyses. The missing data from the N1, N2-P2 was not associated with any condition, rather indicating noise in the EEG and not a systematic bias. Overall, *n* = 29 participants were retained in N1 analyses and *n* = 29 in N2 and P2 analyses.

**Relationship quality**

We employed the seven-item Dyadic Adjustment Scale (DAS-7; Sharpley & Rogers, 1984) to measure relationship quality. The DAS-7 consists of 7 items: three items assess dyadic consensus, three items assess dyadic cohesion, and one item assesses global dyadic satisfaction (the first six items rated on a 6-point scale, ranging from 0 to 5, and the last item rated on a 7-point scale, ranging from 0 to 6). The total score for the DAS-7 is the sum of the responses to the seven items (possible range 0 to 36). The DAS-7 is a well-validated measure (Hunsley, Best, Lefebvre, & Vito, 2001; Sharpley & Rogers, 1984). Prior studies in healthy participants in a current relationship (at least one year) have reported means ranging from 20.9 (*SD*=4.3) to 25.8 (*SD*=4.7), whereas lower means have been reported for participants in clinical settings (*M=*17.8, *SD=*5.5) or participants who are separated/ divorced *(M=*13.4-15.2) (see Hunsley, Best, Lefebvre, & Vito, 2001; Sharpley & Rogers, 1984). In the present sample, Cronbach’s alpha was α=.52, and on average, participants reported high relationship quality/adjustment (*M=*25.84, *SD*=3.18), given the weighting of this mean towards high adjustment. Relationship quality did not correlate with attachment anxiety, Pearson’s *r*=-.06, *p*=.743, or attachment avoidance, Pearson’s *r*=-.03, *p*=.852. We further observed the same pattern of effects as reported in the main text when controlling for relationship quality on our slow versus fast multilevel modelling (see Table S1).

Table S1. Slow versus fast touch: multilevel modeling results for all outcome measures while also controlling for relationship quality as a covariate.

| Effect | Dependent variable | b | *SE* | *p*-value | Confidence intervals | |
| --- | --- | --- | --- | --- | --- | --- |
|  |  |  |  |  | Lower | Upper |
| **Slow touch vs. fast touch** | **N1** | -1.01 | .46 | .029 | -1.92 | -.10 |
|  | **N2** | -2.06 | .76 | .007 | -3.54 | -.57 |
|  | **P2** | 2.85 | .88 | .001 | 1.12 | 4.5 |
|  | **Pain ratings** | .62 | .13 | <.001 | .37 | .87 |
| Attachment anxiety | N1 | -.21 | .53 | .697 | -1.25 | .83 |
|  | N2 | -1.35 | .86 | .114 | -3.03 | .33 |
|  | P2 | 1.54 | 1.15 | .181 | -.72 | 3.80 |
|  | Pain ratings | .01 | .20 | .967 | -.39 | .51 |
| Attachment avoidance | N1 | .01 | .59 | .981 | -1.14 | 1.1 |
|  | N2 | -.68 | .96 | .480 | -2.56 | 1.21 |
|  | P2 | -1.29 | 1.29 | .317 | -3.84 | 1.2 |
| **Attachment anxiety * attachment avoidance** | Pain ratings | -.06 | .22 | .795 | -.48 | .37 |
|  | N1 | -.77 | .79 | .334 | -2.32 | .78 |
|  | **N2** | -2.72 | 1.23 | .027 | -5.13 | -.31 |
|  | P2 | 1.02 | 1.65 | .538 | -2.22 | 4.24 |
| **Touch condition * attachment anxiety** | Pain ratings | -.01 | .29 | .971 | -.58 | .56 |
|  | N1 | 1.16 | .64 | .068 | -.086 | 2.4 |
|  | N2 | 1.26 | 1.05 | .229 | -.79 | 3.31 |
|  | P2 | -2.01 | 1.23 | .101 | -4.41 | .393 |
| Touch condition * attachment avoidance | **Pain ratings** | -.41 | .18 | .023 | -.76 | -.06 |
|  | N1 | .34 | .73 | .639 | -1.09 | 1.7 |
|  | N2 | 2.19 | 1.17 | .061 | -.10 | 4.49 |
|  | P2 | 1.78 | 1.37 | .195 | -.91 | 4.48 |
| Touch condition * attachment avoidance * attachment anxiety | Pain ratings | .15 | .19 | .4#7 | -.22 | .51 |
|  | N1 | .17 | 1.04 | .867 | -1.87 | 2.2 |
|  | N2 | 1.31 | 1.50 | .384 | -1.63 | 4.25 |
|  | P2 | -2.21 | 1.76 | .207 | -5.67 | 1.23 |
|  | Pain ratings | -.26 | .26 | .305 | -.76 | .24 |
| c. Relationship quality | N1 | -.14 | .10 | .182 | -.34 | .06 |
|  | N2 | .06 | .17 | .711 | -.26 | .39 |
|  | P2 | -.35 | .27 | .193 | -.87 | .18 |
|  | Pain ratings | -.02 | .04 | .668 | -.09 | .06 |

**Note.** Significant main effects and interactions are highlighted in bold. Baseline pain as a covariate was statistically significant across all pain outcomes, *p*<.05.

**Interaction between attachment anxiety and attachment avoidance on the N2 local peak amplitude**


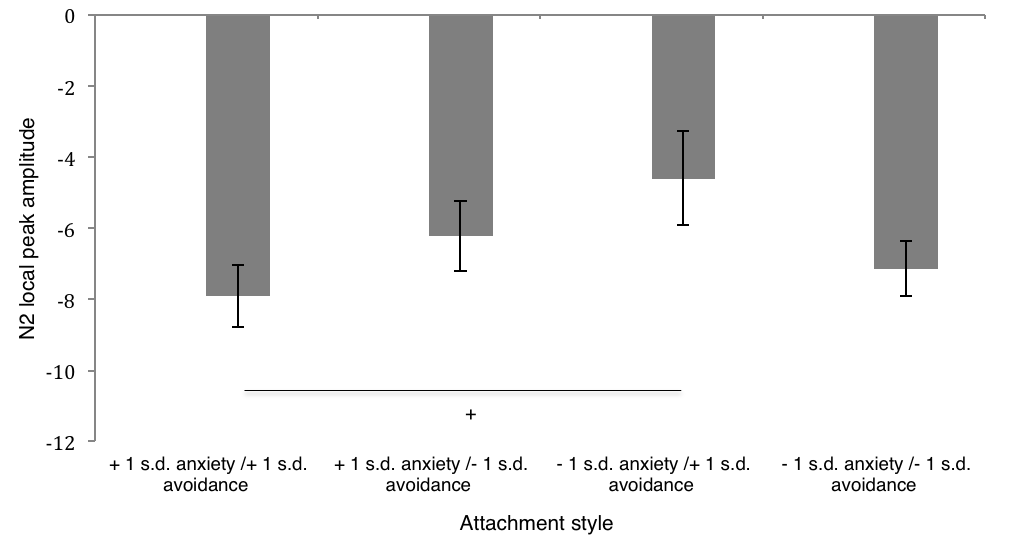


Figure S1. Effects of attachment anxiety by attachment avoidance on the N2 local peak amplitude plotted at low (-1 s.d.) and high (+1 s.d.) attachment anxiety and attachment avoidance scores. Error bars denote SE of the predicted margins. Note. No comparisons were statistically significant / plus sign indicates a trend towards significance between +1 s.d. anxiety/ +1 s.d. avoidance and -1 s.d. anxiety/ +1 s.d. avoidance.

**Attachment style dimensions in our sample**

We compared our attachment anxiety and attachment avoidance scores to those reported as the ECR-R norms (see Fraley, last modified 2012 on The Information on the Experiences in Close Relationships-Revised (ECR-R) Adult Attachment Questionnaire; internal.psychology.illinois. edu/∼rcfraley/measures/ecrr.htm). These norms were obtained from more than 17,000 individuals completing the ECR-R online, with an average age of 27 (*SD*=10). Similarly, we also compared our attachment anxiety and attachment avoidance scores to our earlier work on the effects of affective touch (by stranger) on pain (see Table S2 for descriptive statistics). Our results suggest that our sample reported lower attachment anxiety and attachment avoidance as compared to the general population, *t*=-3.16, *p*=.003, *t*=-8.26, *p*=.001, respectively, as well as compared to our earlier work, *t*=-2.41, *p*=.02, *t*=-2.34, *p*=.02, respectively.

Table S2. Mean (SD) for attachment anxiety and attachment avoidance scores (ECR-R) collected from the current sample, our earlier work on affective touch and the normal population.

|  | Attachment anxiety | Attachment avoidance | N |
| --- | --- | --- | --- |
| The current study | 2.50 (0.75) | 2.55(.69) | 32 |
| Earlier work on affective touch and pain | 2.97(1.01) | 2.97(0.93) | 50 (25 per group) |
| General population | 2.92(1.19) | 3.56(1.12) | 17,000 |

**Correlations between touch pleasantness ratings and related outcomes**

Table S3. Touch pleasantness ratings and pain relation outcomes – correlations results

| Outcome | Stroking velocity condition | Perceived touch pleasantness | Pearson’s r | *p* value |
| --- | --- | --- | --- | --- |
| Pain rating | Slow touch | Pleasantness rating slow touch velocity | -.07 | .715 |
|  | Fast Touch | Pleasantness rating fast velocity | -.02 | .914 |
| N1 | Slow touch | Pleasantness rating slow touch velocity | .06 | .758 |
|  | Fast Touch | Pleasantness rating fast velocity | .15 | .474 |
| N2 | Slow touch | Pleasantness rating slow touch velocity | -.04 | .827 |
|  | Fast Touch | Pleasantness rating fast velocity | -.34 | .075 |
| P2 | Slow touch | Pleasantness rating slow touch velocity | -.13 | .523 |
|  | Fast Touch | Pleasantness rating fast velocity | .21 | .280 |
